# Supplementary material for: Revelations of service-learning project: Multiple perspectives of college students’ reflection
Source: PLoS One. 2021 Sep 23;16(9):e0257754. doi: 10.1371/journal.pone.0257754 (PMC8459965; doi:10.1371/journal.pone.0257754)
Supplement: S1 Appendix — (DOC) [file pone.0257754.s001.doc]

**Appendix 1**

1. What impact does the service-learning experience have on your learning?

2. What do you think about the children, role of teachers and preschool after service-

learning?

3. What do you think about the arrangement of reflection activities in class?

4. Do you have any thoughts on changing things, the world, and yourself after service-

learning?

5. What do you hope you can do in the future?

6. Are there any other issues you would like to talk about?
