# Supplementary material for: Revelations of service-learning project: Multiple perspectives of college students’ reflection
Source: PLoS One. 2021 Sep 23;16(9):e0257754. doi: 10.1371/journal.pone.0257754 (PMC8459965; doi:10.1371/journal.pone.0257754)
Supplement: Attachment — Submitted filename: Response to Reviewers_PONE.doc [file pone.0257754.s002.doc]

**PONE-D-20-32439：Response to Reviewers**

We would like to thank the reviewers for their time and efforts reading our manuscript and providing insightful suggestions for the improvement of our work. We have read the comments carefully, and tried to follow the suggestions as closely as possible. Specific changes in this revision are summarized below.

**For Reviewer #1**

**Q1:**

I recommend to the authors the option of linking the expression ‘service-learning’ with a script, since there is scientific-pedagogical literature that advises it. In my opinion it is a convenient option since it implies an indissoluble union between the terms that best defines the global concept of SL. In any case it is a personal option but, please, authors should be consistent in the entire text.

**A1:**

Thank you for your comment. The term service learning has been revised to service-learning in this manuscript.

**Q2:**

Please, author should make sure that the text follows the journal guidelines (formatting, references, etc.)

**A2:**

Thank you for your comment. The text has been revised in accordance with the journal guidelines.

**Q3:**

In the current version of the abstract, the method is not yet clearly explained. For instance, I would appreciate to know clearer information on the service actions on which the SL program was built.

**A3:**

Thank you for your comment. The supplementary explanation is highlighted in the abstract and stated as follows.

Thirty-six college juniors enrolled in an academic service-learning course were asked to complete a service-learning project, to undertake service activities in the form of eight storytelling sessions at preschools. This storytelling plan was 1.5-hour-long each, twice per month, for 16-weeks.

**Q4:**

In Finally, I strongly advise for a proper specification/description and justification of the data analysis approach and techniques.

**A4:**

Thank you for your comment. The supplementary explanation is highlighted in the abstract and stated as follows.

Qualitative case study approach was employed in this study. Data consisted of documentation of the participants’ reflections, focus group interviews, and research logs. The thematic analysis was employed to analyze data and data were analyzed using the constant comparative technique.

**Q5:**

It would be essential to clarify the gap existent in this field in order to justify the reasons why it is necessary to investigate along these lines. There are several studies on SL in the field of Physical Education Teacher Training that could update and reinforce this foundation on SL reflection outcomes.

**A5:**

Thank you for your comment. The supplementary explanation is in section 1, and the highlight is as follows.

Previous studies of service-learning have shown a positive impact on student learning outcomes [2, 3, 5, 6, 7, 20, 21]. The quality of reflection activities affects learning outcomes related to service-learning [23]. In particular, the quality of reflection focuses on the structure and clarity of reflection practice [24, 25]. By means of group discussion or written reflection activities, students can share their learning experience together [5, 14], make their tacit knowledge explicit [13], and use new cognition in the next service-learning [11].

**Q6:**

The aim of the study should be clearly specified and justified.

**A6:**

Thank you for your comment. The purpose of this research is in the last paragraph of section Introduction, and highlights are as follows.

Accordingly, this study aimed to answer questions regarding the contents of students’ reflections at various stages of the academic SL curriculum. How do reflection contents differ at various stages? What revelations can the analysis of reflection contents yield for service-learning? These research questions may provide insight for college educators to understand the reflection perspectives of students in the SL process and may serve as a reference for the design of SL reflection activities.

**Q7:**

I would like the author to reinforce the final paragraph in page 4 with more references.

**A7:**

Thank you for your comment. The supplement has been added in the final paragraph in page 7, as shown in highlight.

Practical qualities of SL reflection include the following: (a) Connection: this quality emphasizes the inseparability of classroom from community learning, sentimental learning from cognitive learning, and experience from practice. (b) Continuity: SL curricula require continuous arrangement of reflective activities before, during, and after individuals’ service. (c) Context: The service experience and the choice of institutions must be relevant to the students’ learning content to provide students with an integrative learning and service experience. (d) Challenge: Learning occurs when new experience and information conflict with preconceived concepts, using confusion appearing in the thinking process to prompt growth. (e) Coaching: Challenging preconceived concepts is at the core of growth. Students are prone to experience frustration and confusion in the process of developing new perspectives. Instructors must offer an appropriate degree of appreciation and support and must guide students to accommodate new perspectives to process their experience and observation in relation to their current understanding and develop alternative interpretations [17, 18, 31, 40].

**Q8:**

I am surprised that all the participants had previous SL experience. Could this background have conditioned the results somehow? Could you deepen on this issue?

**A8:**

Thank you for your comment. The supplementary explanation is in section 3.2, and the highlight is as follows.

These students had only one-time service-learning experience before, and most of the service content was clerical and cleaning work. The service organization was not in preschool and had no actual contact with children. This is their first experience in serving children.

**Q9:**

In relation to the SL intervention programme carried out, it should be better organized from my point of view.

**A9:**

Thank you for your comment. The supplementary explanation is in section 3.1, and the highlight is as follows.

The service-learning program in this study refers to participants who completed the Preschool Classroom Management course used the knowledge they obtain from the course, including ground rules, classroom discipline, time management and use, and storytelling skills, to undertake service activities in the form of eight storytelling sessions at preschools. The design of this service-learning program is based on Geleta and Gilliam [51], as shown in Table 1.

**Table 1 Preschool Storytelling Service-learning Program**

| **Phase** | **The content of the program** |
| --- | --- |
| **Preparation period** | 1.Understand the needs of preschool  2.Explain the content, implementation method and evaluation of the service-learning plan  3.Provide relevant information and professional knowledge to help students carrying out service activities |
| **Action period** | 1.Visit preschool and establish mutually beneficial partnerships  2.Perform eight storytelling activities for students |
| **Evaluation period** | 1.Share the results of service-learning for students  2.Discuss the process and experience of service-learning for students  3.Vote for the best service award for teacher and students |

**Q10:**

On what theoretical bases was the structure of the focus group interviews supported?

**A10:**

Thank you for your comment. The supplementary explanation is in section 3.4, and the highlight is as follows.

Focus group interviews use interviews in a group context, through the process of group interaction and discussion, to achieve the purpose of collecting information [53].

**Q11:**

Could you better explain the sampling, analysis and saturation processes? In this vein, I think it would be necessary to incorporate references to strengthen the analysis section: qualitative approach, saturation, sampling techniques…

**A11-1:**

Thank you for your comment. The qualitative approach is in section 3.4, and the highlight is as follows.

This research took the thematic analysis approach to analyze and categorize the qualitative data [54].

The researcher first suspended their preperceptions and value judgments to let the data speak for itself and endeavored to uncover meanings in the data [55].

The researcher took a line-by-line analysis approach to data coding, assigned preliminary names through conceptualization, and used the constant comparative method to reveal the attributes and dimensions of concepts [56].

The researcher grouped similar ideas into categories and further categorized them into abstract concepts, which were then developed into the main concepts [55].

**A11-2:**

Thank you for your comment. The saturation processes is in section 3.4, and the highlight is as follows.

The process continues until no new concepts appear, that is, the data is saturated [53], and the core concepts are compiled at this stage [57].

**A11-3:**

Thank you for your comment. The supplementary explanation is in section 3.4, and the highlight is as follows.

This article aims to expand the understanding of the experiences of students’ reflections at various stages of the academic SL curriculum. Therefore, the case study method was adopted [52].

**Q12:**

If possible, the results and discussion section should be completed with more recent and specific references on SL in teacher training.

**A12:**

Thank you for your comment. The supplementary explanation is in sections 4.1.2 and 4.1.3, respectively, and the highlight is as follows.

In agreement with previous studies [3, 6, 22, 22], our data suggested that the service activity in question is combined with the Preschool Classroom Management course, providing the participants with the opportunity to integrate professional knowledge and service experience.

This study is consistent with other research [2, 20, 21, 22, 50], the participants connected the service experience with their mental aspects and attained psychological growth by exploring their own capabilities.

This study support research [3, 6, 20, 21] that the participants began to take an interest in social issues and may be attributed to their experiences of interacting with the preschoolers, who motivated them to think social issues from their professional perspectives.

**Q13:**

There are a lot of subsections in this section. Is it possible to simplify or clarify them?

**A13:**

Thank you for your comment. It has been modified in Section 4.1.2, and highlighted as follows.

When participants began service activities, the content of their reflections shifted toward professional learning and personal growth. In terms of professional learning, P22 grasped the skill of using vocal variations to attract the preschoolers’ attention. She said “When speaking to first-year preschoolers, one needs to slow down. Otherwise, they are unable to understand well, and different characters need to have different voices! I had to exaggerate my voice to draw their attention” (R-1-P22). ~~P8 learned that the classroom ground rules must be established before the storytelling began. She said “…the way to prevent children from becoming distracted or chatting among themselves was to secure an agreement with the children before the storytelling session began that they must remain seated on the floor for the storytelling to continue. If anyone left his or her place, I would stop the story” (R-2-P8).~~ P13 learned to choose the appropriate picture book for preschoolers. She said, “The picture book’s content needs to be suitable for the specific age range. The pictures need to be large, and the plot needs to be interesting enough to keep kids engaged. After these storytelling sessions, I learned that picture book selection was very important!” (F-P13).

Previously, students didn't know that preschoolers’ behavior would be affected by their families. In the process of service-learning, this idea of students has changed. Students’ interpretation and reinterpretation of events in order to make their own experiences meaningful is the key to understanding transforming in viewpoints and thinking habits [23]. ~~After interacting with the preschoolers, the participants noticed how a family environment affected children.~~ P25 said, “After this experience, I found that children are not empty-headed. There was a kid who had a dog at home, and he knew how to take care of it. This may be related to the family environment” (R-3-P25). ~~P9 thought that she learned the ability to observe preschoolers’ behavior. She said, “I led an origami session with the preschoolers. I demonstrated once, and they followed. I observed that their small muscles developed well since they really knew how to work with paper” (R-4-P9).~~

These reflections are connected with the participants’ professional knowledge. ~~This may be because the service activity in question is combined with the Preschool Classroom Management course, providing the participants with the opportunity to integrate professional knowledge and service experience.~~ In agreement with previous studies [3, 6, 21, 22], our data suggested that the service activity in question is combined with the Preschool Classroom Management course, providing the participants with the opportunity to integrate professional knowledge and service experience. Therefore, when instructors are designing service activities, the prior knowledge of students must be taken into account so that students may acquire professional learning through reflection. Just as Eyler [18] observed, context is one of the critical elements contributing to effective SL reflection.

In terms of personal growth, the participants considered storytelling to be an experience of challenging oneself. P19 said: “The second time when I was in the first-year class, I expected that the kids would be unable to sit still, so I talked slowly and used simple sentences to tell the story. The children were really engaged! The best thing is that I succeeded in the challenge!” (F-P19). The storytelling experience allowed them to improve their ability to improvise. P12 said, “There were more disruptions than I had expected during the story-telling session for the children. Asking them to quieten down was not easy. I thought to myself that the hubbub could not go on like that, so I stopped talking. They started to be aware that they might have done something wrong and then settled down” (F-P12). Some participants thought that the service experience brought a sense of accomplishment. P7 said, “After finishing the storytelling session, I would think of the children’s laughter and thought that I must have done something right [because] I can make them happy simply by telling them stories. I feel a great sense of accomplishment” (R-5-P7).

~~Some participants thought that the service experience enabled them to review their career development. P6 said “For some time, I was not sure if it was the right decision for me to choose early childhood care and education as my major, but after interacting with these kids, I found them lovely and decided to teach them well. [The experience] was interesting to me.” (R-5-P6). Accordingly, the participants connected the service experience with their mental aspects and attained psychological growth by exploring their own capabilities [2, 41, 42, 46]. These effects were also essential references for assessing the outcomes of the professional SL curriculum [12].~~

The service-learning experience creates a situation that challenges students’ assumptions and perceptions and stereotypes of others, which leads to a sense of disequilibrium. This sense of disequilibrium requires reflection to help students clarify and critically evaluate personal values, and develop values and attitudes based on new knowledge and experience [24]**.** This study is consistent with other research [2, 20, 21, 22, 50], the participants connected the service experience with their mental aspects and attained psychological growth by exploring their own capabilities.

**For Reviewer #2**

**Q1:**

The introduction gives context and highlights important features of service learning. However, I think it is too specific when describing the theoretical background using Dewey and Kolb’s theories. Instead of that, I would include more information on the context of service learning in Taiwan, particularly with pre-schoolers.

**A1:**

Thank you for your comment. It has been modified in Section 2.2, and highlighted as follows.

In Taiwan, the "University Service-Learning Program" promulgated by the Ministry of Education in 2007, focuses on service-learning integrated with courses, and aims to effectively combine "community service" with "academic courses". Therefore, the application of service-learning courses is widely promoted by university teachers. For teacher educators, service-learning is regarded as a teacher education programs [3, 6, 20]. The service targets of these programs range from preschoolers to high school students. Among them, service-learning for young children is combined with professional courses [5, 21, 22]. These service-learning setting include preschools and zoos [5, 7, 21, 22]. The service-learning content includes planning the preschool learning area and leading children to activities in the learning area [21], as well as learning activities that design activities and lead children to explore nature [5, 22].

The theoretical basis of reflection on service-learning is as follows. (a) Dewey’s experiential learning model: Dewey regarded reflection as the reconstruction and reorganization of experience, a unique problem-solving approach, and a means of learning with which learning outcomes can be achieved [32]. Therefore, learning is a process of generating experiences from reflection in challenging circumstances and converting such experiences into knowledge. (b) Kolb’s experiential learning cycle: Individuals perform reflective learning by establishing a learning cycle of several stages, namely concrete experience, reflective observation, abstract conceptualization, and active experimentation [33]. This theory supports the argument that SL is a type of pedagogy and holds that reflection is a critical factor in theoretical and practical SL experiences. (c) The accordion effect of Sheckley [34]: The two authors considered the processes of reflective judgment and reflective action to be the core of experiential learning. When students’ new experiences and expectations differ, students rethink, reconceptualize, and even change their perspectives to learn from their own experiences despite setbacks and pressures. (d) Social identity development theory: This theory holds that SL participants can only produce cognitive, sentimental, and behavioral changes when they perceive and reflect upon the attitudes of other groups in their own experiences [19]. The aforementioned perspectives demonstrate that student experience and reflection during the SL process interact and produce learning effects; therefore, the process enables students to acquire new concepts and promotes the development of holistic education.

**Q2:**

It is not clear if the research had a Research Ethics Committee approval. It would be worth mentioning if it did and, if not, why did the researcher decide not to have this approval. This is particularly important, as apparently the professor in charge of the course is also the researcher. What are the implications in terms of the pre, during and post activities and data collection strategies?

**A2:**

Thank you for your comment. We have confirmed with our IRB that prospective approval and review was not necessary for our study. This research is a non-human research scope, and it is not a human research subsidized by the Ministry of Science and Technology. Currently, there are no regulations in Taiwan that must be sent to the Research Ethics Committee for review. Based on the principle of informed consent, the researcher explained the purpose and methods of the research to the participants before the research, and invited students to participate in the research to obtain the consent of the participants orally. In addition, the researchers also expressed that they should keep the research data properly and keep the confidentiality principle. In this study, the role of the researcher is to be both a teacher and a researcher. Therefore, the researcher reflects on the unequal relationship between herself and the participants, and faces this fact honestly to gain the trust of the participants with sincerity.

**Q3:**

Table 3 is not clear. Why does it start with 5 and 6 in the first row?

**A3:**

Thank you for your comment. It has been modified, as shown in Table 5.

**Table ~~3~~ 5. Reflection Content and Performance for Different S**tages

| **Stage** | **Reflection Content** | **Performance** |
| --- | --- | --- |
| **Pre-service** | Personal expectations | 1. Increasing practical experience 2. Telling stories well |
| **In-Service** | Professional learning | 1. The skill of using vocal variations to attract the preschoolers’ attention 2. ~~The classroom ground rules to be established before the storytelling begin~~ 3. Learning to choose the appropriate picture book 4. Noticing how a family environment affected children 5. ~~Learning the ability to observe preschoolers’ behavior~~ |
|  | Personal growth | 1. Storytelling to be an experience of challenging oneself 2. Improving their ability to improvise 3. Bringing a sense of accomplishment 4. ~~Reviewing their career development~~ |
| **Post-service** | Social concern | 1. Considering education to be the fundamental basis of the country 2. Pondering on the responsibilities of a teacher 3. Paying attention to the teaching and nurturing of children from disadvantaged groups |

**Q4:**

Results can be presented at the beginning in a table summarising the main themes and subthemes or in a figure. Sometimes it was hard to follow.

**A4:**

Thank you for your comment. The supplementary explanation is in section 4, and the highlight is as follows.

In order to summarize the main themes and subthemes in Section 4, the Table 4 is used and briefly follows.

**Table 4. Perspectives of Participants’ Reflection**

| 1.Reflection Content and Performance for Different Stages | 1-1 Pre-service: Personal expectations |
| --- | --- |
|  | 1-2 In-service: Professional learning and Personal growth |
|  | 1-3 Post-service: Social concern |
| 2.Acquire Active Learning from Service Experience | |
| 3.Different Reflection Activities Trigger  Participants’ Different Reflection Dimensions  and Functions | 3-1 Writing activities focus on personal reflection, allowing for self-examination and adjustment |
|  | 3-2 Group discussion activity focused on dialogue, enabling learning and identity acquisition |
| 4.Students’ Emotions Shift from Anxiety and Fear  to Joy or Disquietude | |

**Q5:**

The results section can benefit of more in-depth analysis. Results could be more specific in terms of the transformation path of the students, and further explain themes such as personal growth, or acquire active learning, for example. These are some of the examples:

a. Personal growth is very descriptive and doesn’t provide an analysis of all the possible links to the student’s responses. The first stage (pre-service) is definitely the moment to set personal expectations, however, it would be worth noting the different types of expectations, if there were individual only, or focused on the collective, for example.

b. In the case of “in service reflection…” theme, this particular part: “After interacting with the preschoolers, the participants noticed how a family environment affected children. P25 said, “After this experience, I found that children are not empty-headed. There was a kid who had a dog at home, and he knew how to take care of it. This may be related to the family environment” (R-3-P25), is an example of context in the SL experience, however it shouldn’t be the only one because this is one of the key aspects of the SL strategy. Later on, during post-service reflections, you mention how students are much more aware of the challenges at the societal level, this is a theme that is worth exploring with more depth to really analyse how the students move from recognising children as thinking humans, to understanding the complexities of the social sphere.

**A5-A:**

Thank you for your comment. The supplementary explanation is in section 4.1.1 and 4.1.2, respectively, and the highlight is as follows.

These expectations include increased practical experience and good performance in storytelling.

The service-learning experience creates a situation that challenges students’ assumptions and perceptions and stereotypes of others, which leads to a sense of disequilibrium. This sense of disequilibrium requires reflection to help students clarify and critically evaluate personal values, and develop values and attitudes based on new knowledge and experience [24]**.**

**A5-B:**

Thank you for your comment. The supplementary explanation is in section 4.1.2 and 4.1.3, respectively, and the highlight is as follows.

Previously, students didn't know that preschoolers’ behavior would be affected by their families. In the process of service-learning, this idea of students has changed. Students’ interpretation and reinterpretation of events in order to make their own experiences meaningful is the key to understanding transforming in viewpoints and thinking habits [23].

The community of student life is different from the children, family and community they come into contact with in service learning [2]. Therefore, the experience of service-learning enables students to examine their own values and limited understanding of children, their families, and communities [58]. The impact on students is a commitment to social problems and social responsibilities [13].

**Q6:**

Conclusions present central issues developed throughout the paper. However, I would suggest to link it much more with the national context, the implications for undergraduate teaching and how this pedagogical strategy serves to improve societal issues, for example.

**A6:**

Thank you for your comment. The supplementary explanation is in section 5.1, and the highlight is as follows.

The findings of this study proved that service-learning can promote students' professional learning, personal development and social concern. This part is consistent with the previously reported results [5, 6, 7, 14, 21]. The implications for higher education in Taiwan, service learning provides college students with a teaching method that connects theory and practice, and it is continuous experiential learning rather than a one-time experience. This kind of experiential learning experience can help students to understand and rethink their role as community members, and critically think and put forward opinions on social issues from their own professional perspective.

**Q7:**

Recommendations can be more specific on specific actions to be taken at different levels.

**A7:**

Thank you for your comment. It has been modified in Section 5.2, and highlighted as follows.

According to the research results, the following suggestions are provided. The suggestion to university teachers is that instructors may design structured reflection activities for the SL curriculum with reference to the various service stages, and provide continuous reflection activities for students throughout the semester. When designing reflection activities, instructors might take into account students’ learning preferences to design reflection activities, such as writing or discussion activities on an individual or a group basis, and accommodate various students’ reflection needs. Instructors should design reflection activities that provide guidance and take into account students’ emotional states. Moreover, appropriate support and feedback should be available to assist students in learning how to address negative emotions during the service process. Suggestions for students, students should actively participate in academic SL courses, accumulate practical experience through "learning in service" and "service in learning", revise and adjust teaching experience from "reflection on action". When students have more opportunities to engage in service-learning, the more likely they are to learn to overcome obstacles.
