# Supplementary material for: Revelations of service-learning project: Multiple perspectives of college students’ reflection
Source: PLoS One. 2021 Sep 23;16(9):e0257754. doi: 10.1371/journal.pone.0257754 (PMC8459965; doi:10.1371/journal.pone.0257754)
Supplement: Attachment — Submitted filename: Response to Reviewers_PONE.doc [file pone.0257754.s003.doc]

**PONE-D-20-32439：Response to Reviewers**

We would like to thank the reviewers for their time and efforts reading our manuscript and providing insightful suggestions for the improvement of our work. We have read the comments carefully, and tried to follow the suggestions as closely as possible. Specific changes in this revision are summarized below.

**For Reviewer #1**

**Q1:**

The article has improved substantially since the last revision I did. In my opinion, the current version of the manuscript almost meets all the elements required for its publication in such hight impact journal. I would only ask the authors that, at this point of the process, it would be convenient to update and reinforece the theoretical framework and discussion section with recent articles (published during the last three or less years) with maximum impact on this topic. The following are suggested. All of them present common features that could slightly enrich the authors approach.

- Marttinen, R., N. D. Daum, D. Banville, and R. N. Fredrick. (2020). “Pre-service teachers learning through service-learning in a low SES school”. Physical Education and Sport Pedagogy, 25 (1): 1-15.

- Chiva-Bartoll, O., Moliner, L., Salvador-García, C. (2020). Can service-learning promote social well-being in primary education students? A mixed method approach. Children and Youth Services Review; 111 pp. 1-8.

- García-Rico, L., Martínez-Muñoz, F., Santos-Pastor, M.L., Chiva-Bartoll, O. (2021). Service-learning in physical education teacher education: a pedagogical model towards sustainable development goals (Epub ahead of print).International Journal of Sustainability in Higher Education; online pp.--

- MacPhail, A., and R. Sohun. (2019). “Interrogating the enactment of a service-learning course in a Physical Education teacher education programme: Less is more?”, European Physical Education Review 25 (3): 876-892.

**A1:**

Thank you for your comment.

**A1-1:**

This paper "Pre-service teachers learning through service-learning in a low SES school" proposed by Marttinen, R., ND Daum, D. Banville, and RN Fredrick (2020) has been published on page 6 of this article, added in p .14, as shown in highlight.

(a) Professional learning: Improve student understanding and practical capabilities in relation to adaptive education [5]; enhance students’ pedagogical knowledge, capabilities of planning and implementing collaborative curriculum, skills of designing crossdisciplinary pedagogical activities, and professional attitudes [7]; help students develop curriculum-design and pedagogical capabilities, gain greater understanding of pedagogical events, maintain favorable instructor–student relationships, develop counseling abilities, enhance management skills, improve interaction skills with instructors, and foster ethical professional values and attitudes [3]; enhance the self-efficacy of student’ teaching technology, the belief in the integration of teaching technology, and understand the role of teaching technology in education [~~43~~ 44]; development of professional identity of pre-service teachers [~~44~~ 45]; and developing classroom and behavior management skills [~~45~~ 46].

These reflections are connected with the participants’ professional knowledge. In agreement with previous studies [3, 6, 21, 22, 46], our data suggested that the service activity in question is combined with the Preschool Classroom Management course, providing the participants with the opportunity to integrate professional knowledge and service experience. Therefore, when instructors are designing service activities, the prior knowledge of students must be taken into account so that students may acquire professional learning through reflection. Just as Eyler [18] observed, context is one of the critical elements contributing to effective SL reflection.

**A1-2:**

The research participants of this article is college students, but the research participants of the paper “Can service-learning promote social well-being in primary education students? A mixed method approach” of Chiva-Bartoll et al. are primary education students (third to fifth grade), so citation is not considered.

**A1-3:**

This paper "Service-learning in physical education teacher education: a pedagogical model towards sustainable development goals" proposed by García-Rico et al. (2021) has been supplemented on p.6, p.7, p.15, as shown in highlight..

(b) Individual Growth: Boost students’ self-worth [~~46~~ 47]; promote personal growth and career exploration [20, 48]; and guide students to perceive positively, identify personal weaknesses, hone communication skills and problem solving abilities, stimulate ideas through peer observation and collaboration, provide collective emotional support, learn to care for others, explore their career, stimulate service dedication, improvise actions according to immediate circumstances, and permit self-reflection [7, 21].

(c) Social Concern: Improve students’ sense of social responsibility and civic engagement [20, ~~47~~, 48, 49]; nurture students’ humanistic and social concerns and allow students to experience ethnic differences and care for the disadvantaged [3, 48]; make students aware of factors affecting preschool education quality [21]; provide students with information regarding social attitudes, leadership skill development, and opinions in the community [~~48~~ 50]; enhance students’ understanding of the importance of community involvement [~~49~~ 51]; and how they can contribute to the community [~~50~~ 52].

This study support research [3, 6, 20, 21, 48] that the participants began to take an interest in social issues and may be attributed to their experiences of interacting with the preschoolers, who motivated them to think social issues from their professional perspectives. The community of student life is different from the children, family and community they come into contact with in service learning [2]. Therefore, the experience of service-learning enables students to examine their own values and limited understanding of children, their families, and communities [~~58~~ 60]. The impact on students is a commitment to social problems and social responsibilities [13]. These social concerns may subsequently be internalized as the participants’ values, and this would help the participants not only to develop professional identities but also to constitute the core values for their future education career [2, 6, 48, ~~59~~ 61].

**A1-4:**

This paper “Interrogating the enactment of a service-learning course in a Physical Education teacher education programme: Less is more?” proposed by MacPhail, A., and R. Sohun. (2019) has been supplemented on p.3, as shown in highlight.

Institutions of higher education across the world have begun actively promoting the SL curriculum in light of the striking results that the United States has achieved in implementing service-learning [27]. Kendall [28] remarked that service-learning has been perceived as a scheme, a philosophy, or pedagogy. SL is a type of pedagogy that achieves an instruction goal, namely connecting community services with educational institutions [6, 23, 29, 30]. It is currently considered a high-impact educational practice ~~[30]~~ [31].
